# Effects of different acute stressors on the regulation of appetite genes in the carp (*Cyprinus carpio* L.) brain

**DOI:** 10.1098/rsos.230040

**Published:** 2023-02-15

**Authors:** Paulina Pawlak, Alexander Burren, Andreas Seitz, Constanze Pietsch

**Affiliations:** ^1^ Agronomy, Bern University of Applied Sciences, Zollikofen, Bern CH-2052, Switzerland; ^2^ Division of Behavioural Ecology, Institute of Ecology and Evolution, University of Bern, Wohlenstrasse 50a, CH-3032, Hinterkappelen, Bern, Switzerland; ^3^ Institute of Natural Resource Sciences, Zurich University of Applied Sciences, Wädenswil, Zürich CH-8820, Switzerland

**Keywords:** aquaculture, stressors, feeding behaviour, gene expression regulation, appetite regulation

## Abstract

Our understanding of the timing of stress responses and specific roles of different regulatory pathways that drive stress responses is incomplete. In particular, the regulation of appetite genes as a consequence of exposure to different stressors has not been studied in sufficient detail in fish. Therefore, a stress trial was conducted with koi carp, aiming at identifying typical effects of stress on regulation of appetite genes. The stressors tank manipulation, air exposure and feed rewarding were chosen. The responses to these stressors were evaluated 10, 30 and 60 min after the stressors were applied. Orexigenic and anorexigenic genes were investigated in four different brain regions (telencephalon, hypothalamus, optic tectum and rhombencephalon). The results show that, apart from the typical appetite regulation in the hypothalamus, the different brain regions also display pronounced responses of appetite genes to the different stressors. In addition, several genes in the serotonergic, dopaminergic and gaba-related pathways were investigated. These genes revealed that rearing in pairs of two and opening of the tank lid affected anorexigenic genes, such as *cart* and *cck,* which were not changed by air exposure or feed rewarding. Moreover, distress and eustress led to limited, but distinguishable gene expression pattern changes in the investigated brain regions.

## 1. Introduction

Aquaculture is increasingly important. Farmed fish are only able to grow optimally if they are fed according to their needs. In addition, it has been shown that appetite strongly determines the digestion of the feed particles [1]. Fish can be trained to fixed feeding times [2] and their appetite is regulated accordingly. On the other hand, external factors such as light quality and temperature affect the feed intake in fish [3,4]. However, the most important factors that impair the appetite of farmed fish are often disease and stress. Stress is known to interfere with the well-being of an animal, which can also include feed uptake and growth performance. Nevertheless, the quality, strength and duration of exposure to a stressor determine how the fish reacts, i.e. whether the stressor is appraised as a negative or a positive stimulus. While negative stimuli affect the well-being of an animal [5], eustress as positive stimulus can also lead to higher locomotory activity, e.g. with respect to feed anticipatory activity [6], and is therefore considered a stressor as well. Chronic negative stress, in particular, is known to affect appetite regulation and growth performance in teleosts (e.g. [5,7,8]), but research on appetite regulation in the brain has focused mainly on the hypothalamus [9].

The present study thus focused on the effects of different stressors on gene expression patterns in the carp brain. Stress in fish is often thought to mainly induce increased levels of the stress hormone cortisol in the blood stream, and it has been shown that an acute or chronic increase in cortisol levels is a potent anorectic effector in fish [10]. However, before stress hormones are released into the blood stream, a number of genes, e.g. belonging to the so-called hypothalamus–pituitary–interrenal (HPI) axis are differentially regulated in the brain [9,11]. Namely, genes analysed in studies interested in investigating the activation of the HPI axis often include neurotransmitters, signal mediators and receptors, such as proopiomelanocortins, corticosteroid releasing factors (crf) and glucocorticoid and mineralcorticoid receptors [12–15].

However, the question remains as to which brain region is most involved in the response of appetite genes to stress. The hypothalamus is the typical centre of appetite regulation, but evidence is accumulating that crosstalk with other brain regions is also important for appropriate regulation of appetite and feeding behaviour. Similar to higher vertebrates, the amygdala in fish also communicates with other brain parts, e.g. the hypothalamus or the brainstem, in order to achieve the behavioural and physiological outputs [16,17]. It has already been shown in rodents that a number of appetite-regulating neuropeptides, namely neuropeptide Y (npy [18]), orexin (ox [19]) and the cocaine and amphetamine-related transcript (cart [20]) at least partially regulate feeding via interactions with crf neurons, while other feeding regulatory factors, e.g. melanocortins, may act independently of crf-related factors [21]. Similarly, npy presence has also been confirmed for the telencephalon, the optic tectum and the rhombencephalon of common carp [22,23].

Nevertheless, the common hypothesis is that the hypothalamus plays an important role in energy homeostasis and appetite regulation in fish. Molecules that reflect the peripheral energy status include ghrelin (ghr), leptin and insulin, and nutrients. These lead to the activation or inhibition of distinct neurons in the feeding centre. Activated or suppressed neurons then result in adjustments to behaviour and metabolism. Proopiomelanocortin (pomc) neurons are major satiety neurons in the hypothalamic arcuate nucleus in mice [24], but only a small portion of the pomc neurons is responsive to sweet-tasting and thus to potentially rewarding molecules, such as sucralose and glucose [24,25].

Appetite regulation generally differentiates two main directions: appetite increasing mechanisms which are called orexigenic, and appetite-reducing pathways leading to anorexigenic effects. The orexigenic potential in the hypothalamus of fish is exerted by agouti-responsive protein/npy neurons, which lead to increased feed intake, whereas the anorexigenic potential involves the activation of pomc/cart neurons, which result in decreased appetite upon activation. An interaction of the appetite-regulating pathways and stress responses has been observed in a number of studies which will be reviewed in the following sections. For example, decreased food intake during stress might be caused by the connection of npy neurons and crf neurons [26,27]. In goldfish (*Carassius auratus*) forebrains, dose-dependent increased *npy* mRNA levels were found after cortisol administration [27]. In zebrafish (*Danio rerio*) stressed by handling, increased *npy* mRNA expression was observed in the hypothalamus [28]. Similar results have been obtained for rainbow trout reared under high stocking densities [29,30]. By contrast, neither short-term crowding nor handling stress resulted in similar changes in tilapia, *Oreochromis mossambicus* [31]. In addition, other brain regions in fish also exhibit different gene expression patterns due to exposure to stress. For instance, intra-peritoneal administration of cortisol in tilapia for 1 day resulted in decreased *npy* mRNA expression in the telencephalon but not in the hypothalamus [32]. By contrast, increased *npy* mRNA expression in the preoptic area and forebrain was influenced by the social status of rainbow trout [33].

Furthermore, it is known that cortisol has a negative feedback effect on crf and urotensin I in the forebrain [34] and may thus reverse anorexigenic effects of crf [35]. However, feeding may also be influenced by cortisol via other neuroendocrine pathways. For instance, prolonged moderate increases in plasma cortisol promoted food intake in goldfish and decreased the expression of *npy* and *crf* in the forebrain, while feeding higher doses of cortisol resulted in decreased *crf* mRNA expression*,* without affecting food intake, and no changes in *npy* mRNA expression were observed [27]. Moderate increases in plasma cortisol over a period of 6 days promoted feed intake in trout [36], while higher plasma cortisol levels decreased feeding activity [37]. Furthermore, chronic administration of cortisol reduced the feed intake in channel catfish, *Ictalurus punctatus* [38]. Finally, glucocorticoids also appear to act directly or indirectly on intestinal nutrient uptake which also affects the appetite, for example, in salmonids [39–41].

Appetite research has also concentrated on other appetite-regulating factors in fish. Oral administration of anorexigenic factor cholecystokinin (cck) antagonists increased food consumption in trout as well as in sea bass [42,43]. Therefore, cck was assumed to be a neuropeptide with important anorexigenic and satiety functions in fish [43–47]. On the other hand, fasting can affect *cck* mRNA expression, for example in the brain and intestine of winter flounder (*Pleuronectes americanu*s), yellowtail (*Seriola quinqueradiata*) and zebrafish [44,48,49]. However, fasting in cavefish for 10 days did not change whole-brain *cck* expression [50], whereas intra-peritoneal injection of cavefish, *Astyanax fasciatus mexicanus*, with mammalian ghr resulted in increased feed intake. An extracerebral role of cck in regulating digestive processes in common carp was also suggested by the study of Kuzmina [51]. *Ghr* expression in the brain showed the typical pattern of an orexigenic neuronal factor in grass carp, *Ctenopharyngodon idellus* [52], and in line with that ghr injection studies stimulated feed intake in goldfish [53,54]. However, in tilapia (*Oreochromis mossambicus*), chronic administration of tilapia ghr increased food intake, while acute intra-peritoneal injections did not [55]. Different results have been obtained for trout in which intra-peritoneal injections of trout ghr had no effect on feed intake, and central injection of trout ghr decreased feed intake 1 h after the injection [56,57]. These results confirm that species-specific differences exist in relation to the effects of ghr on feed intake, which also vary with the origin of ghr, the type of administration and exposure time.

With respect to the anorexigenic factors, *cart*, was originally identified by acute administration of cocaine or amphetamine to rats [58,59], and its name was derived from these observations. Cart is also an anorexigenic factor in fish, for example in goldfish and zebrafish [60–62], and shows low expression values during fasting periods and high expression after re-feeding in carp [63]. Interestingly, the expression of *cart* was not changed by injections of different neurotransmitters into the brain of cavefish [47]. Nevertheless, acute stress increased the *cart* and *pomc* mRNA levels in zebrafish [28], and an increase in *pomc* expression was also observed in the fish used in the present study [11]. However, no changes or even decreased levels were described in rainbow trout exposed to chronic crowding stress [29,30]. Decreased *pomc* expression was also observed in sole reared under high stocking density [64,65]. These results lead to the assumption that, apart from species-specific differences, the complex response of appetite and stress genes depends on the type of stressor.

Several other appetite-regulating genes are known which often interact with each other. For example, orexins increase locomotor behaviour and feeding in fish [66–68]. Interestingly, injection of *cck* into the brain of cavefish resulted in decreased apelin levels in the brain, while apelin injections elevated the *th*, *mtor* and *ox* mRNA expression in the cavefish brain [46]. An ox injection increased tryptophan hydroxylase expression, whereas administration of ghr induced *ox* expression in the brain [47]. The well-known orexigenic neuropeptide in fish, ghr, also increased levels of *npy* and *ox* in goldfish [69,70]. In addition, ox administration has been shown to regulate blood glucose levels goldfish and also the extracerebral expression of gene involved in the glucose metabolism [71]. However, interaction with crf neurons can cause ghr to exert anorexigenic effects in fish [72]. The effect of ghr on the regulation of food intake in fish is thus appraised differently depending on the fish species, the individual life stage and how feed is administered. In trout and tilapia, increased cortisol levels lead to lower circulating ghr levels [32,73]. By contrast, stress increased *ghr* mRNA expression and its receptors in the telencephalon and the preoptic area in the brain of tilapia [31]. Similarly, Cortés *et al*. [28] reported elevated *ghr* levels in the brains of zebrafish after handling stress. Ghr has thus been assumed to decrease the feed intake in fish under chronic stress, but acute stress may have no effect on *ghr* levels and feed intake since other central regulation mechanisms can affect the appetite under short-term stress conditions [31]. One example of counter-regulatory mechanism in response to the anorexigenic effect of ghr involved increased *npy* mRNA expression and agouti-related protein in the hypothalamus in parallel to ghr in zebrafish [28]. Interestingly, *ghr* is also one of the genes that has also been linked to changes in growth hormone regulation in goldfish [74,75], which allows linking the appetite regulation with a growth response. Connection of appetite regulation and growth hormone expression have also been reported for the gastrin-releasing peptide (grp) as well as for cck [75,76].

Nevertheless, stress is also known to affect the activity of the dopaminergic and serotonergic networks in fish, especially following social stress [15,77] which may further affect the appetite regulation in fish. For example, in trout (*Onchorhynchus mykiss*), acute stress resulted in activation of monoaminergic networks in the forebrain [78,79]. Furthermore, it has been shown that personality of European sea bass, *Dicentrarchus labrax* and gilthead sea-bream (*Sparus aurata*) is characterized by certain serotonin levels in the brain, which may also have consequences for individual stress responsiveness in fish [80,81]. Likewise, it was not surprising that the serotonin precursor tryptophan interferes with stress-induced anorexia in rainbow trout [5]. Furthermore, there is increasing evidence demonstrating that serotonergic and dopaminergic pathways exert anorectic effects in fish, for example, as changes in monoaminergic activity due to the exposure to antidepressants [82]. Serotonergic activity in fish also leads to increased *pomc* expression, decreased expression of orexigenic neuropeptides, such as *npy* and agouti-related peptide, and increased expression of anorexigenic neuropeptides including *cart* [83,84]. Inhibitory effects of serotonin administration on feeding activity have also been described for common carp [85]. The ratio of serotonin to its main metabolite, 5-hydroxyindoleacetic acid, together with the blood glucose levels, is obviously important for appetite regulation [84]. Activation of the *5ht2c*-like receptors in the hypothalamus, in particular, was assumed to mediate the inhibitory effect on feed intake in rainbow trout [86–88]. *In vitro* treatment of the telencephalon with noradrenaline and serotonin (5-ht) stimulates the release of crf in tilapia [89]. Serotonergic cell bodies have been found in the raphe nuclei and in several hypothalamic nuclei [90–92], whereas dopaminergic cell bodies appear to be located, for example, in the locus coeruleus in the rhombencephalon [93]. The effects of dopamine appear to be less clear and dependent on the variable expression of hypothalamic neuropeptides. For instance, the administration of dopamine decreases gene expression of the agouti-related protein but not of *pomc* and *npy* [94]. However, oral administration of the dopamine precursor L-dopa inhibits feed intake in sea bass, elevates *npy* expression and has no effects on the expression of the agouti-related protein and *pomc* in the hypothalamus [95]. In the same study, dopamine was observed to induce *crf* expression in the hypothalamus. In addition, there is a correlation between *npy* and *crf* gene expression in subordinate trout [33].

When goldfish are not fed for 7 days, they show increased mRNA expression of the tryptophan and the tyrosine hydroxylase *(th*), which are the enzymes that limit the synthesis of 5ht and dopamine [96]. Mice with a *th* knockout have been shown to be hypophagic [97,98]. In cavefish, fasting also induced *th* mRNA expression in the brain [50]. Furthermore, peripheral injections of both apelin and ox, but not ghr and cck, into the cavefish brain induced significant increases in *th* mRNA abundance. *Th* mRNA abundance in the brain increased after fasting and was further elevated 1 h after feeding in cavefish [50]. Furthermore, lungfish ox-positive cells have been found in close proximity to th-positive neurons [99], which is in line with the anatomical and structural interactions between central catecholamines and ox, ghr and cck that have been reported for mammals [100–102]. This suggests an important role of th in regulating appetite and feeding in vertebrates.

The mechanistic target of rapamycin (mtor) is a serine–threonine protein kinase that plays a central role in nutrient sensing and energy status regulation [103–105]. To this end, mtor-controlled signalling pathways regulate many physiological functions of the nervous system [106]. Rodents show decreased activity in the mtor pathway during fasting periods in contrast with increased activity after feeding [107,108]. In mammals, there is evidence of interactions between mtor and several orexigenic and anorexigenic factors, including npy and cart [109], cck [110] and ghr [108,111]. Consequently, mtor regulation is increasingly also gaining in importance in research on fish. In zebrafish, fasting decreased *mtor* expression in the liver [112]. In addition, *mtor* expression in cavefish brains was significantly increased by ghr injections but was not affected by ox or cck injections [47].

In addition, gamma-aminobutyric acid, gaba, is a neurotransmitter that plays a role in how animals experience anxiety, fear and stress. Accordingly, gaba was observed to stimulate *crf* gene expression in the telencephalon of goldfish [113]. The effects of gaba are mediated by gaba receptors. Gaba receptor a (gaba*_A_*) is important for the development of the brain in zebrafish [114], but has also inhibitory effects on the steroid-dependent release of growth hormones in goldfish [115]. In addition, crosstalk between gaba_A_ and ox has been observed in goldfish [116]. It is therefore not surprising that an interaction between gaba-nergic signalling and feeding behaviour has been identified in fish [117].

The hormone isotocin is known to be involved in social behaviours and aggression in fish [118–120]. In addition, stress also influences isotocin levels, including air exposure, confinement, disturbance, high density, food deprivation or rapid osmotic challenge, which are accompanied by aggressive acts [121–123]. In mRNA expression studies in fish, the precursor molecule (isopre) is typically assessed.

Finally, the opioid system in vertebrates plays a central role in nociception and analgesia, but also in stress responses and reward processing [124]. Food reinforcement, in particular, is based on the activity of the opioid system, which was the reason for also investigating the expression of the opioid receptor delta (opiod) in this study.

In the present study, the koi carp were trained to receive a feed reward at a fixed feeding time, and the effects of different stressors on appetite gene regulation were investigated. In addition, the studies also included analyses of the expression of some genes in the monoaminergic networks. The effects on the HPI axis in these fish have been discussed in a separate study [11]. The present study makes an important contribution towards further understanding the regulation patterns of appetite genes under different stress conditions in fish.

## 2. Materials and methods

### 2.1. Rearing conditions

As described previously [11], 70 juvenile koi carp (*Cyprinus carpio*) were reared in a 290 l tank for two months under optimal conditions and fed four times daily at a feeding rate of 2–3% body weight per day with a commercial diet (Aller Classic with 30% crude protein and 7% crude fat, purchased from Emsland Aller Aqua, Golssen, Germany) prior to the start of the stress experiments. All experimental procedures have been approved under permission number ZH-062–17 by the relevant cantonal veterinarian authorities of Zurich (Switzerland). The fish were trained to receive a feed reward with thawed mosquito larvae (purchased from Zoo Roco, Lyss, Switzerland) for several weeks. As previously described [11], for the stress experiment the fish were: A) taken directly from the rearing tank and sampled for brain and blood (C0), or B) kept in 50 l aquaria for 3 days, continuing the feed rewards and using curtains around the aquaria to prevent any influences caused by the researchers during routine work. After acclimatization, the fish were exposed to three different scenarios: for controls (= C), the curtains in front of the tanks were opened and the lid of the aquarium was lifted and closed again; the feed reward group (= F) received a ration of thawed mosquito larvae, while the animals in the distress group were exposed to air (= A) for 1 min by netting. For each treatment, six individual fish with a total mean weight of 79 g body weight were used. After the individual treatment, the animals remained undisturbed in the aquaria for a further 10, 30 or 60 min. Following this, the fish were anaesthetized with greater than 150 mg tricaine methanesulfonate (MS-222, Sigma-Aldrich, Buchs, Switzerland) per litre and sampled immediately. Plasma parameters have been reported previously [11]. The brains were cut into four regions (tel = telencephalon, hyp = hypothalamus, opt = optic tectum, rhomb = rhombencephalon).

### 2.2. Polymerase chain reaction conditions

The polymerase chain reaction (PCR) conditions have been reported previously [125]. In short, gene expression studies by means of qPCR on a LC480 Light Cycler II from Roche (Basel, Switzerland) were performed separately for the four different brain parts. Prior to this, all genes were validated, the respective PCR products confirmed by sequencing, as well as two to three reference genes for each brain region, as explained earlier [11]. The target genes included orexigenic as well as anorexigenic genes (the primers are listed in electronic supplementary material, table S1). All gene expression values have been calculated relative to the expression of the selected reference gene (ΔCt method) and were further calculated as fold-changes compared with the respective controls.

### 2.3. Calculations and statistics

A principal component analysis (PCA) was run in R studio, and the PCA results were limited to the first two components for the present study. The two-component analysis explained a mean variance of 84.0% in the telencephalon, 67.5% in the hypothalamus, 67.4% of the variance in the optic tectum and a mean variance of 64.4% in the rhombencephalon. The cos^2^ values derived from the PCA were used to prepare heatmaps for each dataset. Data modelling was carried out in R studio, as described previously [11]. The R codes and all raw data have been added to the electronic supplementary material.

## 3. Results

### 3.1. Effects of tank manipulation

**Figure 1.**
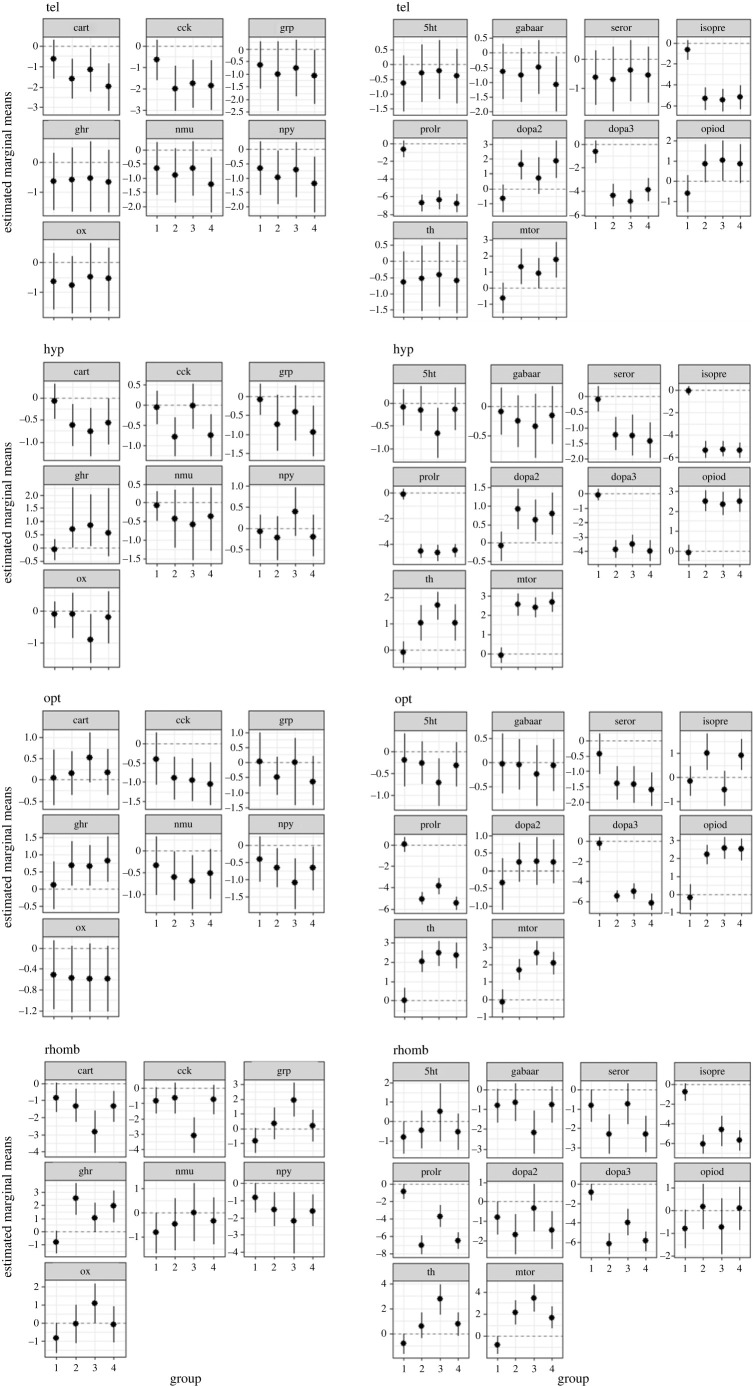
Estimated marginal means in each of the four brain parts in the control fish 0 (group 1), 10 (group 2), 30 (group 3) and 60 min (group 4) after the different treatments, mean ± credible intervals; models based on *n* = 6 per treatment.

Figure 1 shows the differences in gene expression between the control animals after 0, 10, 30 and 60 min. For *cart,* mRNA expression in the tel was significantly (*p* < 0.05) lower in the C10 and C60 groups compared with C0. The *cck* mRNA expression in C0 was higher than in all other control groups in the same brain region. Similarly, the C0 group showed higher *cck* expression in the hyp, but only compared with the C10 and the C60 groups. In addition, the *cck* expression in the hyp was also higher in the C30 group compared with the C10 and the C60 groups (*p* < 0.05). Moreover, mRNA expression of *cart* in the hyp was only found to be higher in the C0 group when compared with the C30 group (*p* < 0.05). The *cart* and *cck* expression in the opt was not found to be significantly different. In the rhomb, only the C30 group showed lower *cart* and *cck* expression levels compared with all other control groups.

No differences were found in the expression of *grp, ghr, nmu, npy, ox* in the tel and in the opt, and *nmu* and *npy* expression also revealed no significant differences in the rhomb. However, the expression of *grp* in the hyp was significantly decreased in the C60 group compared with the C0 group, and *ghr* expression was found to be higher in the C30 group than in the C0 group (*p* < 0.05). In the rhomb, there was a significant increase in *grp* expression in the C30 group compared with the other control groups, as well as a difference between the C0 group and the C10 group, where the expression of *grp* was lower in the C0 group. In the same brain region, the expression of *ghr* was found to be lower in the C0 group than in the three other control groups (*p* ≤ 0.002) and also lower in the C30 group than in C10 (*p* < 0.05).

The expression of *nmu* and *ox* was unchanged in the hyp, but *npy* expression was lower in the C10 group compared with the C30 group (*p* < 0.05). Moreover, *ox* expression was higher in the rhomb in the C30 group, when compared with both the C0 and the C60 groups.

No difference was found for *serotr* expression in the tel between the controls. By contrast, there was a significant decrease in *serotr* expression in the hyp and in the opt in the control groups, when compared with the C0 group (*p* ≤ 0.02). In the rhomb, *serotr* expression was higher in the C0 group compared with the C10 and the C60 groups, and expression in both the C10 and the C60 groups was significantly lower than in the C30 group. A pronounced difference in *isopre*, *mtor* and *prolr* expression (*p* < 0.001) was observed between the C0 group and all the other control groups in the tel, hyp, as well as in the rhomb, where *isopre* and *prolr* expression in the C0 group was higher and *mtor* expression was lower compared with the other control groups. This pattern was also observed for *prolr* and *mtor* expression in the opt; however, *prolr* expression was observed to be higher in C30 compared with the C10 and C60 in the opt. *Mtor* expression was also higher in C30, but compared only with C10. In the rhomb, an increase in *mtor* expression was also observed in the C30 group compared with both the C10 group and the C60 group (*p* < 0.001). In addition, *isopre* expression in the opt appeared to be similar, but there was no significant difference in expression between the C0 and the C30 groups. However, *isopre* expression was lower in the C0 and C30 groups, when compared with both the C10 and the C60 groups. The expression of *mtor* was also lower in the tel in C30 than in C60 (*p* < 0.05). In the tel, as well as in the hyp, expression of *dopa2* and *dopa3* was significantly higher and lower, respectively, after 10, 30 and 60 min of sham treatment compared with the grouped animals in C0, while this pattern was only observed for *dopa3* and not *dopa2* in the opt and the rhomb. Expression of the *dopa2* gene in the rhomb was higher only in the C30 group, when compared with the C10 group. Interestingly, the expression of *dopa3* was also significantly higher in the tel in C60 than in C30, but lower in the opt and the rhomb (*p* < 0.05). Expression of *opiod* was lower in C0 than in the other control groups in the tel, hyp and opt. A similar pattern was observed in the rhomb, but the difference to the C30 group was not significant. No difference in the expression of *th* occurred in the tel, but a significant increase in the expression of this gene was observed in the hyp, in the opt and in the rhomb in C10, C30 and C60, when compared with the C0 group (*p* ≤ 0.004). In addition, *th* expression was also increased in the hyp and in the rhomb in the C30 group compared with C10, and also in the rhomb, when compared with C60.

In the rhomb, only the C30 group showed a lower *gabaa* expression level compared with all other control groups, while mRNA expression of this gene was not significantly different in the other brain regions.

**Figure 2.**
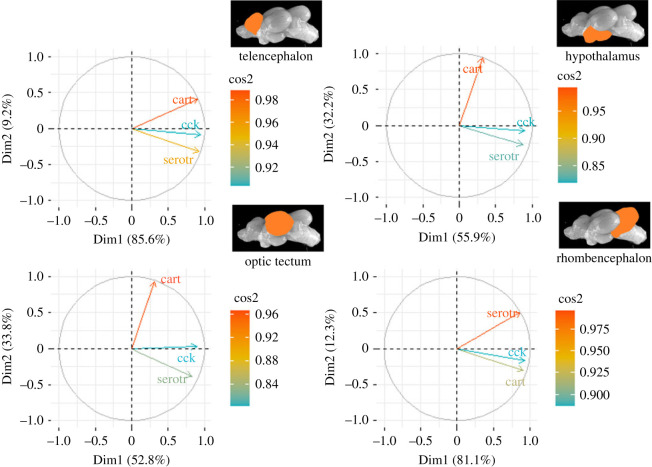
Quality of representation of the mRNA expression of *cck*, *cart* and *serotr* in control fish 0, 10, 30 and 60 min after tank manipulation on the factor map cos^2^ (the numbers next to Dim1 and Dim2 indicate the percentage of the variance in the datasets that is explained by the first two components of the PCA), *n* = 6 per treatment.

Finally, and compared with the effects on gene expression in distressed and feed-rewarded animals, genes in control animals that appeared to be unique for the tank manipulation scenario were *cck, cart* and *serotr,* which were therefore also used for a separate PCA. The first two components for each PCA were used to prepare heatmaps of the cos^2^ values for each brain region (figure 2). This analysis confirmed that the three selected genes showed a number of strong loadings on both components. The two-component PCA explained 94.9% of the total variance in the tel, 88.1% in the hyp, 86.6% in the opt, and 93.4% of the total variance in the rhomb. Interestingly, the highest cos^2^ values for the tel, hyp and opt were observed for *cart*, whereas *serotr* was top-ranking in the rhomb.

### 3.2. Effects of distress

**Figure 3.**
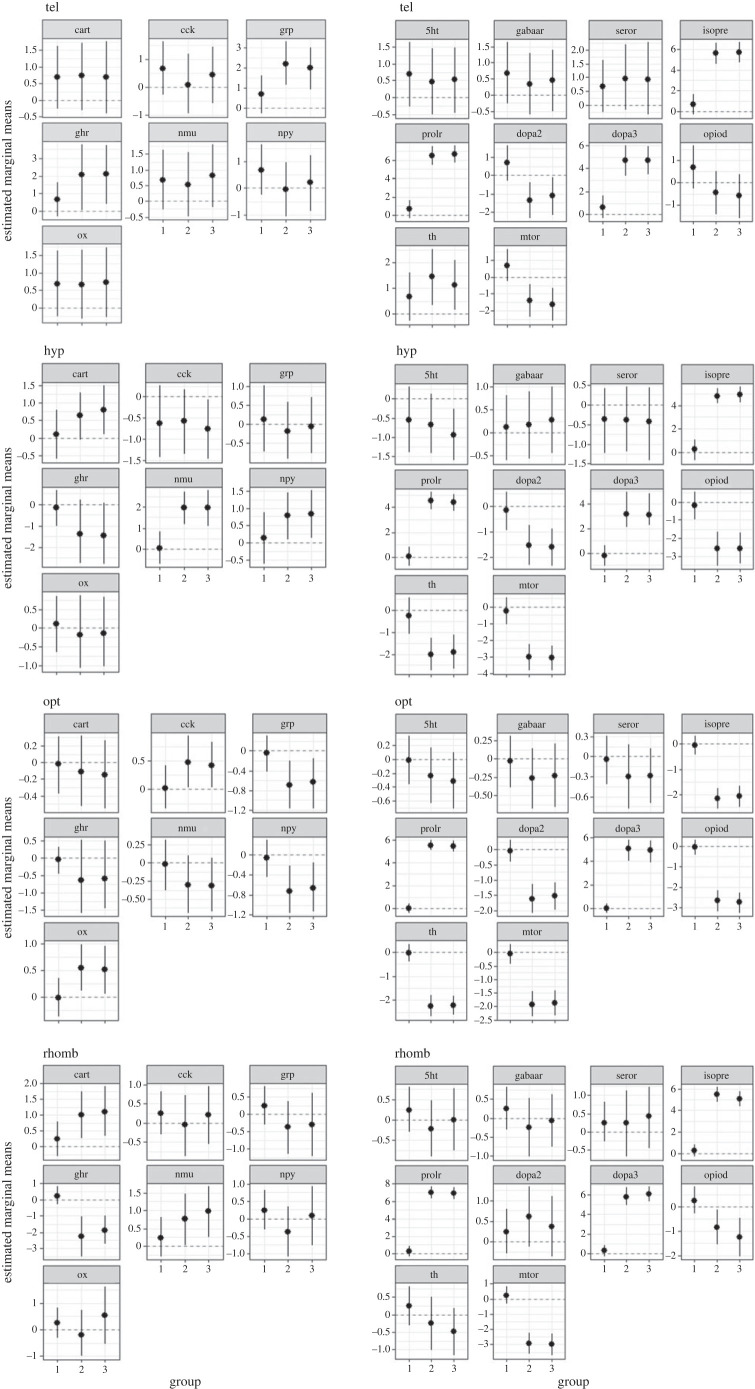
Estimated marginal means in each of the four brain regions in the control fish 10 min after the different treatments, mean ± credible intervals; models based on *n* = 6 per treatment, group 1 = C10, group 2 = F10, group 3 = A10.

**Figure 4.**
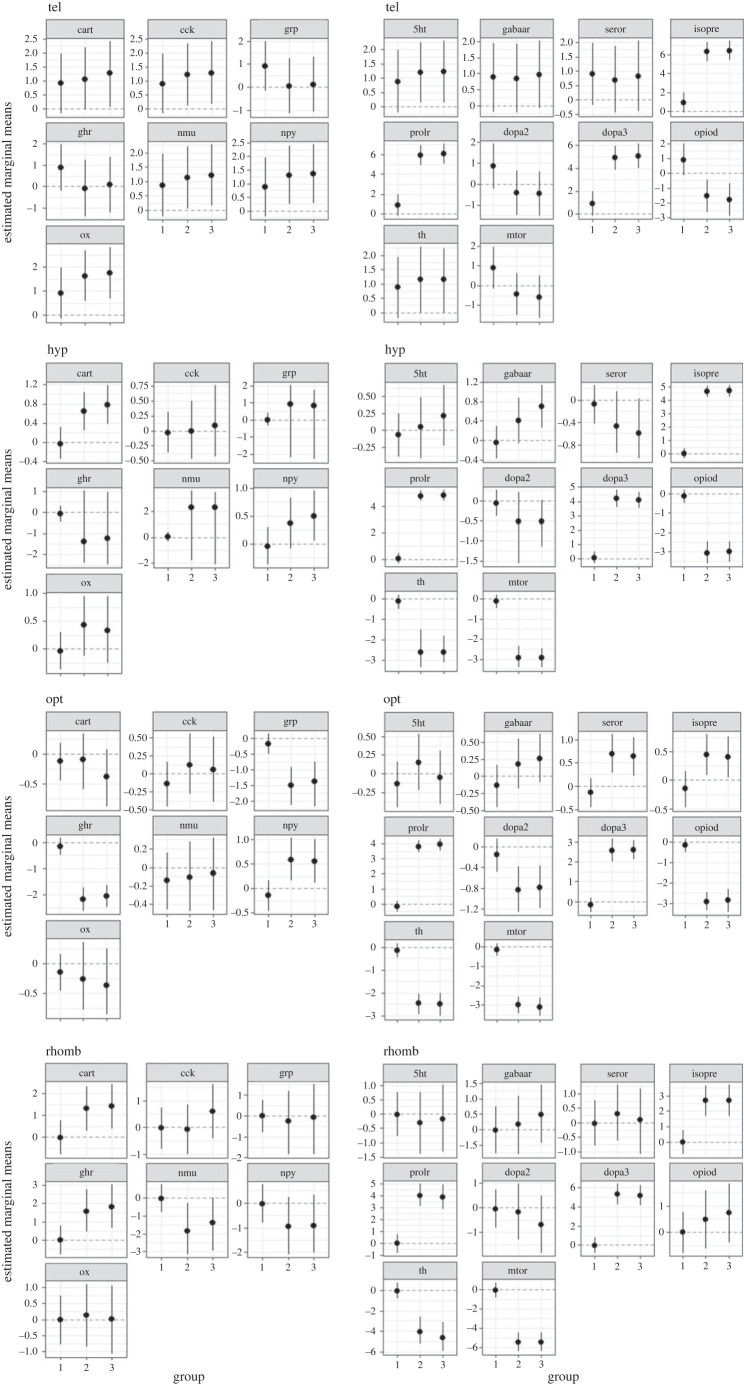
Estimated marginal means in each of the four brain regions in the fish 30 min after the different treatments, mean ± credible intervals; models based on *n* = 6 per treatment, group 1 = C30, group 2 = F30 and group 3 = A30.

In all four brain regions that were investigated, *isopre*, *prolr* and *dopa3* were increased compared with the control group, except in the opt, where *isopre* was decreased 10 and 60 min after both treatments. In each brain region, the expression of *opiod and mtor* were lower in the 30 and 60 min groups compared with the control group, except for the rhomb 30 min after the treatment, where the expression of *opiod* increased in both treatment groups. The analyses also showed that 10 min of air exposure did not influence *cart*, *cck, gabaa, ghr, nmu, npy, ox, serotr* and *th* mRNA expression in the tel, but air exposure caused a significant decrease in *grp* and *dopa2* expression compared with the C10 group (figure 3). In the hyp, *dopa2* and *th* expression were lower than in the relevant control group and C10, and *nmu* was higher in both treatment groups. In the opt, *grp* expression was significantly lower than for the control exposures (*p* < 0.05). In the same brain region, *npy, dopa2* and *th* expression, as well as the expression of *ghr* in the rhomb, were also significantly lower 10 min after the treatment compared with the control animals. At the time point, 30 min after the treatment, all four brain regions shared the influence of the air exposure on the increased expression of *isopre, prolr* and *dopa3*. In addition, *dopa2* and *opiod* expression were significantly lower in the tel than in the control group (*p* < 0.001). In the hyp, *cart, isopre, prolr* and *dopa3* expression were found to be higher than for the control exposures; however, the expression of *opiod* and *th* was found to be lower. In contrast with feed rewarding, only air exposure affected *gabaa* (*p* = 0.006) and *npy* (*p* < 0.05) in the hyp 30 min after the treatment (figure 4), by increasing their expression. When looking at the opt, a decrease in *grp, ghr, dopa2, opiod* and *th* expression and an increase in the expression of *npy* and *serotr* occurred compared with the C30 group. In the rhomb, there was a significant increase in the expression of *cart* and *ghr* and a decrease in *th* expression compared with the C30 control group.

**Figure 5.**
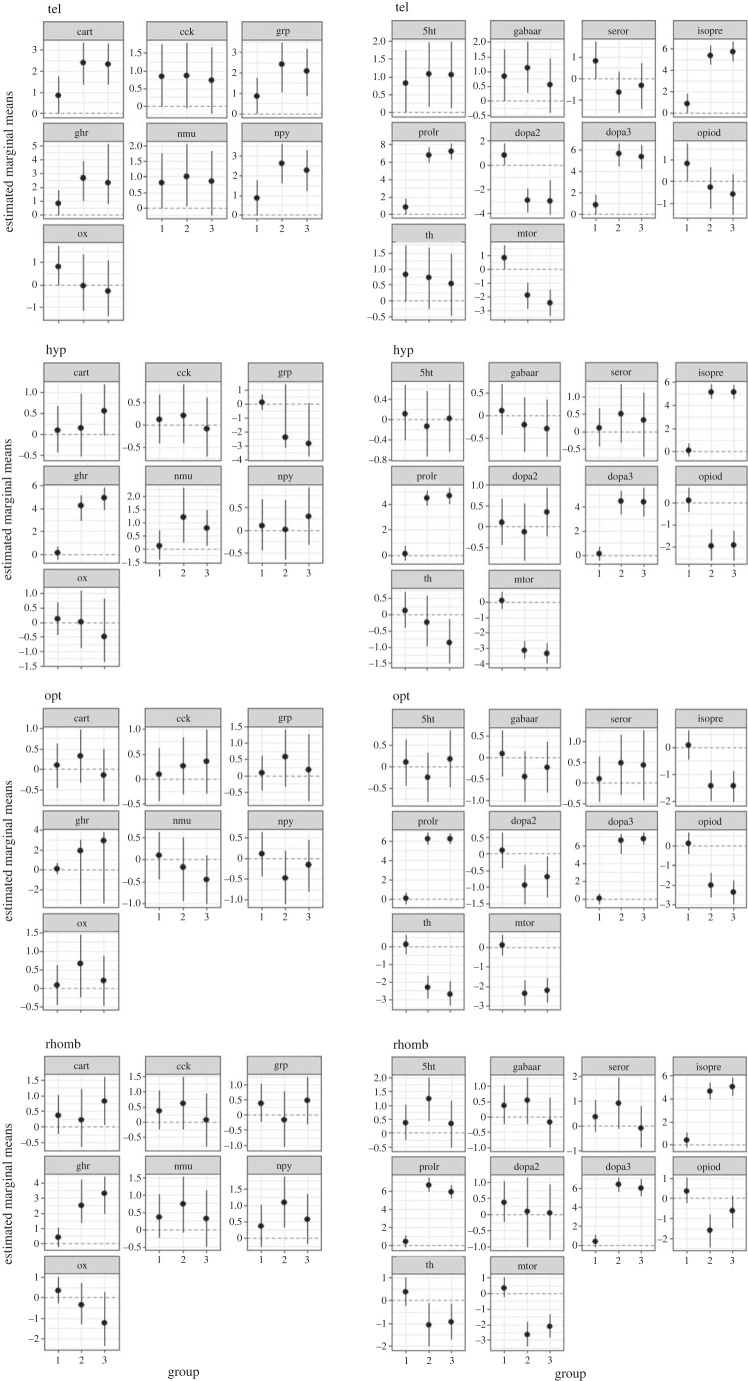
Estimated marginal means in each of the four brain regions in the fish 60 min after the different treatments, mean ± credible intervals; models based on *n* = 6 per treatment, group 1 = C60, group 2 = F60 and group 3 = A60.

Waiting for 60 min after the treatment revealed higher *cart, npy, isopre, prolr* and *dopa3* expression (figure 5)*,* as well as a lower expression of *serotr, dopa2, opiod* and *mtor* in the tel compared with the C60 control group. In addition, at the same time point, *gabaa* expression in the tel was significantly higher in the feed reward group than in the air exposure treatment group. In the hyp, a significant increase in *ghr, isopre, prolr* and *dopa3*, as well as a decrease in *opiod* and *mtor* expression were observed compared with the respective gene expression levels in the C60 group. In contrast with the feed reward group, air exposure caused a significant decrease in *grp* and *th* expression in the hyp compared with the C60 group (*p* < 0.05). In the opt, decreased expression was observed only in *isopre, dopa2, opiod, th* and *mtor* 60 min after air exposure*,* whereas an increase in expression was seen for *prolr* and *dopa3* compared with the levels in the C60 group.

In the rhomb, there was a lower level of *ox* expression compared with the control (*p* < 0.05), which was not observed when the levels in the feed reward group were compared with the C60 group. In addition, 60 min after the treatment *serotr* expression in the rhomb was significantly lower in the air exposure group compared with the animals that had received a feed reward. Furthermore, the expression of a number of genes in the rhomb, which included *ghr, isopre, dopa3* and *prolr,* exhibited an increase compared with the expression levels in the control group*.* The genes *opiod*, *th* and *mtor*, however, showed a decrease in expression in the same brain region as the control group.

### 3.3. Effects of eustress

Similar to what was observed for air exposure, compared with the control group, feed rewarding increased *isopre*, *prolr* and *dopa3* expression and decreased *opiod* and *mtor* expression 10 min after the treatment in all four brain regions that were investigated. However, a decrease of *isopre* expression was also observed in the opt. In the hyp, only *nmu* expression was significantly higher and expression of *dopa2* and *th* was lower in both treatment groups compared with the relevant control group C10 (figure 3). In the opt, *grp* expression was significantly lower than for the control exposures (*p* < 0.05). The expression of *ghr* in the rhomb was also lower 10 min after feed rewarding. In the hyp, *dopa2* and *th* expression were also significantly lower 10 min after the treatment compared with the control animals, but *npy* expression was significantly higher. In contrast with the exposure to air, only feed rewarding increased *ox* expression in the opt 10 min after the treatment (*p* < 0.05).

At the timepoint 30 min after the treatment (figure 4), only *dopa2* and *opiod* expression were also significantly lower in the tel than in the control group (*p* < 0.001). In the hyp, *opiod* and *th* expression were found to be lower than for the control exposures, whereas the expression of *cart* was higher in the rhomb, and there was a significant increase in expression of *cart* and *ghr* and a decrease in *th* expression compared with the control group C30. In addition, *nmu* expression in the rhomb was decreased in the feed reward group, but was not significantly influenced by air exposure.

Waiting for 60 min after the treatment revealed an increase in the expression of *cart, npy, isopre, prolr* and *dopa3* in the tel compared with the C60 control group (figure 5), with a decrease in the expression of *serotr, dopa2, opiod* and *mtor*. In addition to the above-mentioned difference in the *gabaa* expression in the tel between the air exposure group and the feed reward group, there was a significant increase in *grp* and *ghr* expression in the feed reward group compared with the C60 group (*p* < 0.05). In the hyp, significant increases in *ghr, isopre, prolr* and *dopa3* expression were observed compared with the respective gene expression levels in the C60 group; however, the expression of *opiod* and *mtor* was decreased. In contrast with the group exposed to air, feed rewarding caused a significant increase in *nmu* expression in the hyp compared with the C60 group (*p* < 0.05). In the rhomb, 60 min after feed rewarding, *5htr* exhibited a significant increase compared with the expression levels in the C60 group as well as in the air exposure group. A number of genes also exhibited different expression levels, including *ghr, isopre, dopa3* and *prolr* with increased expression in the rhomb compared with the control group*,* and decreased *opiod, th* and *mtor* expression. The expression of *opiod* in the rhomb was significantly lower in the F60 than in the A60 group—in contrast with *prolr*, for which expression was higher in the feed reward group than in the air exposure group.

### 3.4. Principal component analyses

**Figure 6.**
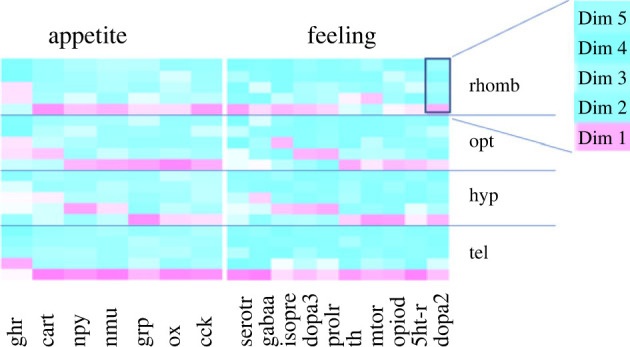
Heatmap profile of the cos^2^ values for five dimensions from the each PCA of (*a*) the appetite-related genes (*ghr, cart, npy, nmu, grp, ox* and *cck*), (*b*) feeling-related genes (*serotr, gabaa, isopre, dopa3, prolr, th, mtor, opiod, 5htr* and *dopa2*) in each of the four brain regions in the control fish 0, 10, 30 and 60 min after the different treatments, *n* = 6 per treatment.

**Figure 7.**
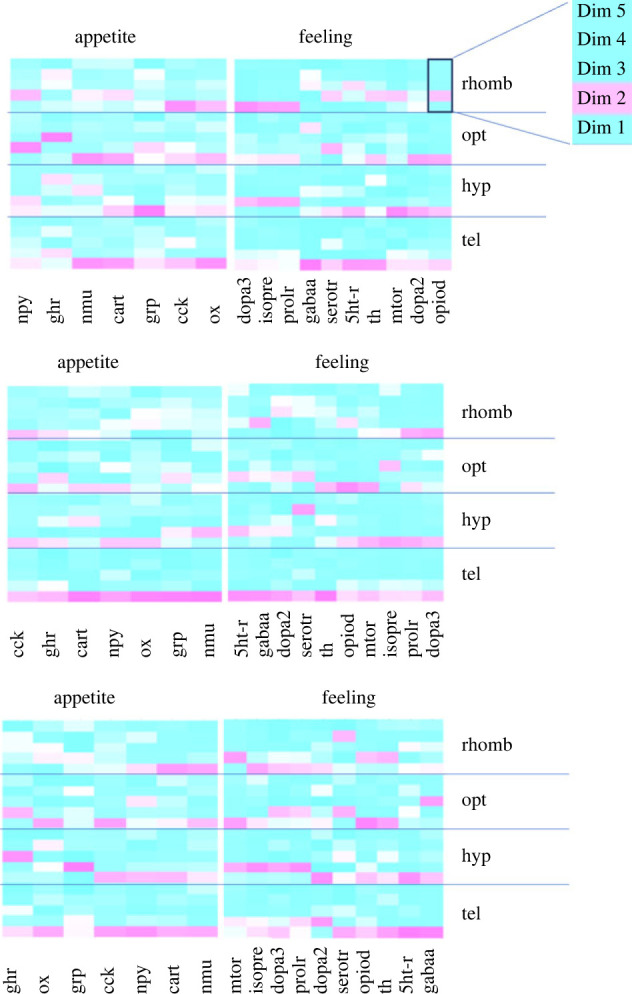
Heatmap profile of the cos^2^ values for five dimensions from the each PCA of (*a*) the appetite-related genes (*ghr, cart, npy, nmu, grp, ox* and *cck*), (*b*) feeling-related genes (*serotr, gabaa, isopre, dopa3, prolr, th, mtor, opiod, 5htr* and *dopa2*), in each of the four brain regions in the fish treated for 10, 30 and 60 min, *n* = 6 per treatment.

In order to reveal the presence of regulation patterns in the genes that were investigated, PCA analyses were conducted once again. The PCA analyses revealed that the first two components that were selected for the PCA calculations exhibited a number of strong loadings on both components. For the appetite-related genes, the two-component PCA explained 84.7% in the tel, but only 59.7% in the hyp, 75.5% of the total variance in the opt, and 74.4% of the total variance in the rhomb (table 1). Similarly, 10, 30 and 60 min after the treatments, the tel showed the highest values for the total variance explained in the datasets (table 2). Furthermore, the first five components for each PCA were used to prepare heatmaps of the cos^2^ values for each brain region (figure 6 and 7).

**Table 1.** Results from the PCA with the first two components with the highest eigenvalue in the telencephalon (tel), hypothalamus (hyp), optic tecum (opt) and rhombencephalon (rhomb) for the four different control groups (C0, C10, C30 and C60). The percentage of variance in relation to the total variance in the datasets that is explained by the individual components (variance exp.) is shown. The gene sets that have been used for the PCA include (I) appetite genes (*cart, cck, grp, ghr, nmu, npy* and *ox*), and (II) serotonergic, dopaminergic and gaba-related genes, summarized here as ‘feeling genes’ (*5htr, serotr, gabaa, isopre, dopa2, dopa3, opiod, prolr, th* and *mtor*), *n* = 6 animals per control group.

component	tel		hyp		opt		rhomb	
	1	2	1	2	1	2	1	2
*appetite*								
eigenvalue	5.041	0.892	2.180	2.001	3.896	1.389	4.006	1.216
variance exp.	72.0	12.7	31.1	28.6	55.7	19.8	57.2	17.4
*feeling*								
eigenvalue	7.233	1.518	4.139	3.292	4.248	2.521	5.313	1.860
variance exp.	72.3	15.2	41.4	32.9	42.5	25.2	53.1	18.6

**Table 2.** Results from the PCA with the first two components with the highest eigenvalue in the telencephalon (tel), hypothalamus (hyp), optic tecum (opt) and rhombencephalon (rhomb) for the three different treatment groups (C, F and A) 10, 30 and 60 min after the treatment. The percentage of variance in relation to the total variance in the datasets that is explained by the individual components (variance exp.) is shown. The gene sets that have been used for the PCA include (I) appetite genes (*cart, cck, grp, ghr, nmu, npy* and *ox*), and (II) serotonergic, dopaminergic and gaba-related genes, summarized here as ‘feeling genes’ (*5htr, serotr, gabaa, isopre, dopa2, dopa3, opiod, prolr, th* and *mtor*), *n* = 6 animals per control group.

component	tel		hyp		opt		rhomb	
	1	2	1	2	1	2	1	2
10 min treatment								
*appetite*								
eigenvalue	3.995	1.052	3.035	1.573	3.081	1.821	2.319	1.914
variance exp.	57.1	15.0	43.4	22.5	44.0	26.0	33.1	27.3
*feeling*								
eigenvalue	4.642	1.544	3.088	2.301	2.814	1.832	3.183	2.100
variance exp.	66.3	22.1	44.1	32.9	40.2	26.2	45.5	30.0
30 min treatment								
*appetite*								
eigenvalue	5.273	0.867	2.959	1.160	3.048	1.401	2.108	1.591
variance exp.	75.3	12.4	42.3	16.6	43.5	20.0	30.1	22.7
*feeling*								
eigenvalue	7.391	1.558	4.574	1.907	3.729	2.682	2.898	2.160
variance exp.	73.9	15.6	45.7	19.1	37.3	26.8	29.0	21.6
60 min treatment								
*appetite*								
eigenvalue	4.569	0.870	2.887	1.597	3.323	1.289	2.754	1.438
variance exp.	65.3	12.4	41.2	22.8	47.5	18.4	39.3	20.5
*feeling*								
eigenvalue	5.673	2.727	4.196	3.369	4.317	2.305	3.793	3.208
variance exp.	56.7	27.3	42.0	33.7	43.2	23.0	37.9	32.1

**Figure 8.**
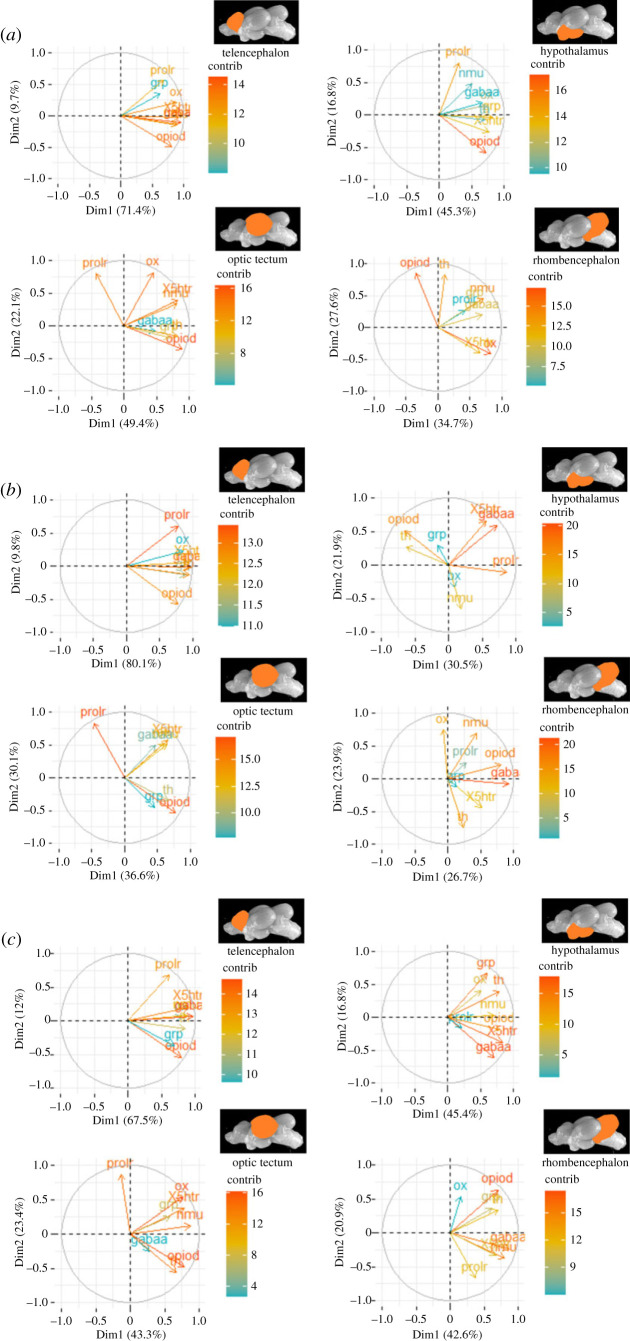
Quality of representation of the mRNA expression of *ox, nmu, gabaa, prolr, opiod, 5htr, grp* and *th* in fish 10 (*a*), 30 (*b*) and 60 min (*c*) after treatment on the factor map cos^2^ (the numbers next to Dim1 and Dim2 indicate the percentage of the variance in the datasets that is explained by the first two components of the PCA), *n* = 6 per treatment.

The genes that exhibited differences in expression between the fish receiving feed rewards and those exposed to air at any of the time points (10, 30 or 60 min) were used for a separate PCA (figure 8). Based on this, the two-component PCA conducted on the 10 min dataset explained 81.1% of the total variance in the tel, 62.2% in the hyp, 71.5% in the opt and 62.3% of the total variance in the rhomb. Similarly, the values for the variance explained in the PCA were 89.9%, 52.4%, 66.7% and 50.6% for the tel, hyp, opt and rhomb, respectively, in the 30 min dataset. Moreover, in the 60 min dataset, the PCA explained 79.5% of the total variance in the tel, 62.2% in the hyp, 66.7% in the opt and 63.5% of the total variance in the rhomb. Interestingly, the highest cos^2^ values for all brain regions taken together were observed for *gabaa, opiod* and *prolr*.

## 4. Discussion

### 4.1. Tank manipulation

Isolated animals commonly need to shift their priorities from foraging to alertness or potential escape, since being separated from their group may make them more susceptible to the risk of predation. It is known that social isolation is not only associated with increased aversive behavioural responses, but also with reduced positive-valence behaviours such as feeding [126]. For larval zebrafish, raising the group size was paralleled by an increased food intake per animal [127]. For the same species, increased activity in the preoptic area was observed as a result of long-term social isolation at least at juvenile life stages, and brain regions that respond strongly to isolation also showed serotonergic activity [128]. The fish in the present study were expected to be hungry at the time of sampling, since the trained feed reward was not given to any of the control fish prior to sampling. Zebrafish deprived of food for a period of 2 h show increased appetite [129,130]. Similarly, it would be expected that the control fish in the present study which were trained to receive a feed reward early in the morning would show signs of frustration and hunger, since they were not fed on the sampling day, even though the tank was opened before sampling of the fish. This is best reflected by the increased *ghr* expression, e.g. in the hyp.

Isolation decreases the appetite in fish, but this effect could not be counteracted by visual cues. Instead, water-borne cues (e.g. water derived from conspecifics) can reverse the appetite inhibition, probably via an isotocin-dependent pathway in the brain [127]. The expression of *isopre* was reduced in most of the brain regions of the tank-manipulated animals (C10, C30 and C60) compared with the control fish in the group tank (C0). Isolation and feed deprivation not only influenced *isopre* expression in the fish, but also affected *cck* and *cart* expression. An earlier study by Wee *et al*. [127] indicated that, at minimum, ablation of isotocin functions affects the nocifensive behaviour. More evidence for appetite-regulating roles of the oxytocin system, which is the same as the isotocin system in fish, can be found in studies using higher vertebrates: insatiable appetite and strong obesity have been linked to impaired oxytocin signalling [131,132] and blocking oxytocin neurons can increase the feed intake [133]. Moreover, defects in the development of oxytocin neurons can cause hyperphagia and obesity [134,135]. However, the complete absence of oxytocin signalling may also be detrimental to feeding [127]. The activation of isotocin pathways in fish by social isolation may represent a negative valence state, similar to the effects described in isolated zebrafish [127]. However, further changes in isopre expression have also been observed in the animals receiving feed rewards and the fish exposed to air. For future studies, investigation of the expression of the receptors for isotocin would also be recommended, in order to improve the evaluation of the activity of the isotocin pathway.

Even so, it must be assumed that there are overlapping behavioural effects of other neuropeptides in the same, relevant brain regions [136] and it is therefore likely that isotocin is not mediating social stress behaviours alone. The activation of other neurons, such as those expressing *crf*, would be necessary to overrule socially dependent influences on behaviour.

### 4.2. Effects of distress

Distress interferes with appetite and feed intake in fish. It is assumed that distress silences the hypothalamic glucosensing, and appetite regulation is then independent of the glucose levels in the blood stream [137,138]. In addition, the expression of the orexigenic neuropeptide *npy* decreased in hyperglycaemic control fish, while the anorexigenic factors *cart* and *pomc* increased. This was not observed in hyperglycaemic fish exposed to crowding stress [29]. In the present study, circulating glucose levels were increased 10 min after the treatment in the groups receiving feed rewards and exposed to air [11]. Normally, anorexigenic factors would be expected to increase and orexigenic factors to decrease in stressed animals with normal circulating glucose levels, leading to a decreased appetite after both a meal and a stressor. Moreover, the expression of *crf* increased in stressed trout independently of the blood glucose levels and influenced the glucosensing capacity in the hyp [30]. Under stress conditions, *ex vivo* administration of *crf* interferes with glucosensing mechanisms in the hyp thereby controlling food intake [139]. However, *crf*-related peptides also modulate feed intake by interacting with the neuronal circuit in the brainstem, which further affects gastrointestinal motility [140,141] via the parasympathetic system, for example in rodents [142]. Whether *crf*-related peptides play similar roles in the rhomb in fish is not known. In our previous study, *crf*-1 mRNA abundance in the rhomb showed downregulation in the C10 group compared with C0, was upregulated 30 min after the tank manipulation and downregulated again in the C60 group [11]. The upregulation of *crf* receptor 1 and 2 was observed in the rhomb 60 min after exposure to air, when compared with the feed reward group.

Proopiomelanocortin (pomc) neurons are major satiety neurons in the hypothalamic arcuate nucleus in mice [24], but only a small portion of the pomc neurons is responsive to potentially rewarding, sweet-tasting molecules, such as sucralose and glucose [25,26]. Our previous study indicated that rearing carp in pairs lowered *pomc1* expression in the tel, hyp and rhomb, but not in the opt, compared with fish reared in a group [11]. In addition, the same study showed that *pomc1* was upregulated in the tel, hyp and rhomb after feed rewarding, but also after air exposure. This indicates that *pomc1* plays an evident role in acute stress responses. However, to date there is no clear evidence of direct involvement in appetite regulation in fish.

Several types of stressors have already been investigated in fish models, including handling, isolation, predator exposure, chemicals or crowding and have been related to increased activity in central monoaminergic systems [78,79,143–145]. The response of the serotonergic system to stress is especially consistent, and serotonin appears to be commonly involved in stress responses. For instance, Gesto *et al*. [78] observed a very rapid increase of serotonergic activity (within seconds) in the forebrain of trout after they were chased. Similar to other stress markers, such as plasma catecholamines and cortisol, the serotonergic activity reaches basal levels again several minutes or hours after stress application, depending on the severity of the stress treatment. Similar patterns can be seen in the current study, where *serotr* mRNA expression increased in the opt 30 min after air exposure; however, it decreased in the tel as early on as 60 min after the treatment. High and sustained serotonergic activity has been observed in chronic stress situations [146–148]. Whether or not these are also responsible for prolonged behavioural changes and reduced growth performance in fish exposed to chronic unpredictable stressors [8] remains to be elucidated for carp.

Clearly, the interaction between serotonin and cortisol in fish is not yet fully understood. For example, Gesto *et al*. [78] assumed that rapid serotonin responses after the application of an acute stressor trigger the activation of the HPI axis in rainbow trout. In addition, the influence of elevated cortisol levels on *5htr* function is mediated by glucocorticoid receptors in fish, and the stimulation of *5htr* by addition of an agonist also resulted in *crf* release in the hyp and acth formation in the pituitary of toadfish, *Opsanus beta* [149]. This may confirm that serotonin transfer induces a cortisol response in this fish species. However, the studies by Ferrari *et al*. [80] and Höglund *et al*. [81] indicated that the personality of sea bass leads to certain serotonin levels in the brain, which subsequently also influence the effect size of stress responses when an acute stressor is used. Nevertheless, dopaminergic activity can also be increased after stress in trout [78] which indicates an involvement of dopaminergic pathways, but does not yet explain the exact mechanisms of action involved.

A high number of behavioural outputs can be influenced by *mtor*-dependent pathways. The appetite-regulating factor, leptin, can induce prolactin mRNA expression via *mtor* and *erk*-1/2 pathways in the goldfish pituitary [150]. In the present study and our previous study [11], the expression of the prolactin receptor was investigated in parallel to the expression of *mtor* and *erk1/2*. While feed rewarding induced *erk1/2* mRNA expression [11] and the *prolr* expression reported in the present study, *mtor* expression was found to be decreased. The regulation of *prolr* expression may occur due to a feedback mechanism of prolactin itself. However, prolactin is widely involved in the regulation of growth and development, as well as the regulation of brain functions and feeding behaviours in vertebrates [151], and prolactin regulation is thus connected to a variety of other regulatory pathways. The release of prolactin is under the inhibitory control of dopamine in teleosts [152,153]. In addition, short-term food deprivation increased dopamine levels in tench, *Tinca tinca* [154]. Control of prolactin levels is therefore thought to be important to supply energy during starvation and exercise in carp [155]. Hence, autocrine or paracrine regulatory mechanisms of prolactin levels appear to be possible, which is also supported by a study using zebrafish embryos [156]. Since prolactin signalling is also connected to dopaminergic pathways, it is noteworthy that a previous study on zebrafish reported that boldness determines the expression levels of dopamine D2 receptor expression [157]. The stressors that were applied to the fish in the present study more often influenced the expression of *dopa3* than *dopa2*. It is therefore assumed that both receptor subtypes have independent functions in fish.

However, other genes are also connected with *mtor*-related pathways. Glyceraldehyde-3-phosphate dehydrogenase (*gapdh*) is a key regulator of *mtor* expression [158]. Interestingly, smaller fish appear to have enhanced levels of *gapdh* expression, suggesting that the *mtor* activity might be suppressed in smaller fish as observed in other species [159]. The investigations by Buller *et al*. [160] also suggest that enhanced *gapdh* expression suppresses *mtor* activation. Accordingly, *mtor* levels were found to be lower in dwarf Arctic charr (*Salvelinus alpinus*) compared with fish showing more advanced growth performance [159]. However, Kocmarek *et al*. [161] indicated that *gapdh* levels may also increase with increasing body mass since more glycolytic muscle mass (i.e. white muscle tissue) is present compared with the entire body mass in larger fish [162]. As described in our previous publication, downregulation of *gapdh* was observed in the rhomb, showing significant differences in fish in C10 and C60 compared with C0 [11]. However, upregulation of *gapdh* was also observed in the opt at 10 min after both treatments, whereas the expression of this gene decreased in the same brain region and in the hyp at 30 min compared with the control group. It is therefore possible that the downregulation of *mtor* after 10 min occurred at least partly due to *gapdh* regulation.

In mammals, it is known that *crf* receptors and actions of the different *gaba* receptor subtypes are connected. For example, the blockade of presynaptic *crf2* receptors decreases *gaba_A_* receptor-mediated inhibitory transmission and increases excitatory transmission through the decreased activation of presynaptic *gaba* receptors that regulate glutamate release. By contrast, postsynaptic *crf1* receptors activate protein kinase A to increase the transmission at specific glutamate receptors [163]. In mice, fasting resulted in a higher *gaba* content in the hyp [164]. In the present study, *gabaa* expression increased in the hyp 30 min after air exposure, which might also be related to the fact that these fish had remained unfed before being treated and sampled. Hence, the food deprivation may have interfered with the stress responses to the air exposure. A contradictory result was observed after 60 min in the tel of fish receiving a feed reward, where *gabaa* was also upregulated.

Distress caused by manipulations has been shown to increase the expression of *npy* in the hyp of zebrafish [28]. Similarly, an increase in expression of this gene was observed in the tel in goldfish after the application of cortisol [27]. This effect is also seen in this study, where the hypothalamic expression of *npy* was increased in the air exposure group already 30 min after the treatment. Furthermore, our previous study [11] shows that plasma cortisol levels were also increased in the air exposure group at the same time point.

Intra-peritoneal injection of ghr, cck and ox in cavefish (*Astyanax fasciatus mexicanus*) has already indicated that on the brain expression level, *th, mtor, cck, ox, apelin* and *cart* interact in a complex network [50]. Injections with ox increased *th* expression and ghr injections induced *mtor* and *ox* expression in the brain. In the same study, *cart* expression was not affected by any of the injection treatments. The results of the present study suggest that *th* and *mtor* are downregulated and the expression of the factors *cck*, *ox* and *ghr* are differentially regulated upon exposure of koi carp to air.

### 4.3. Effects of eustress

The addition of an isotocin receptor antagonist increases food intake in socially isolated zebrafish [127], whereas receptor agonists suppressed food intake in fish kept in a group. In the present study, socially isolated carp fed with the feed reward at the expected time of the day showed increased *isopre* expression, but this characteristic was similar for the animals exposed to air. Therefore, the valence of animals cannot be distinguished based on this marker alone.

Amygdala function and, in particular, the capacity for emotional learning requiring *gabaa* expression, appeared to be dependent on the presence of *neuroD2* in mice [165]. *Neurod* expression in our fish revealed differences in expression 30 min after the treatment in the rhomb between fish exposed to air and the feed reward group, or 60 min after treatment in the opt [11]. By contrast, *gabaa* expression in the tel was found to be different between the groups receiving a feed reward and exposed to air.

In rodents, the gene *mtor* is a serine/threonine kinase found in two functionally distinct complexes, *mtorC1* and *mtorC2,* which are differentially regulated by a great number of nutrients, such as glucose and amino acids, energy (oxygen and ATP/AMP content), growth factors, hormones and neurotransmitters. Two *mtor* complexes are also known in fish [166,167]. *Mtor* controls many basic cellular functions in fish, while an increase in the expression of *mTORC1* complex genes in the skeletal muscle after feed restriction has also been reported [166]. Consequently, *mtor*-dependent pathways may also have been involved in the gene regulation that was observed in the present study.

Fasting was observed to decrease *nmu* expression in the hyp in orange-spotted grouper (*Epinephelus coioides*) and, furthermore, its levels increased 3 h after feeding [168]. In the present study, *nmu* expression decreased in the rhomb after 30 min in the feed reward group. However, after an initial increase in expression in the hyp as early on as 10 min after both treatments, it was also upregulated after 60 min, but only in the feed reward group. This appears to be a typical characteristic after feed intake in fish. The anorexigenic function of *nmu* is further supported by the observation that central injection of *nmu* inhibits locomotor behaviour and feeding in goldfish [169].

The opioid system in higher vertebrates is known to be connected to reward processing [124]. However, it is well known that the different opioid receptor isoforms in fish act independently. In addition, the expression of opioid receptors in fish may be influenced by exposure to stress. For example, when added to the water of larval sea-bream, naloxone, an opioid receptor antagonist, reduced the effects of overcrowding, low pH, high temperature and salinity [170]. The delta type of the receptor has not been related to social behaviour in higher vertebrates [171]. By contrast, our present results from the PCA suggest that the expression of the *opiod* receptor may be indicative of the stress status of the fish.

## 5. Conclusion

Evidence is accumulating that there is a complex brain network through which different components of the stress axis may interact with the mechanisms involved in the regulation of appetite and feed intake. Our results indicate that the potentially appetite-reducing effects of isolation may be related to changes in the expression of *cck* and *cart*. Feed rewarding and distress are perceived differently in carp. The PCA revealed that when the difference between distress, eustress and the controls is evaluated, the genes *gabaa*, *opiod* and *prolr* show the highest impact on the outcome of the PCA. This indicates that, despite the already known involvement of the HPI axis in stress responses, other pathways are also contributing to an appropriate response to different stimuli.

## Data Availability

All data and codes have been added to the electronic supplementary material [172].
